# Thyroid Hormone-Regulated Expression of Period2 Promotes Liver Urate Production

**DOI:** 10.3389/fcell.2021.636802

**Published:** 2021-04-01

**Authors:** Xiaoting Chen, Mian Wu, Nan Liang, Junxi Lu, Shen Qu, Haibing Chen

**Affiliations:** ^1^Department of Endocrinology and Metabolism, Shanghai Jiao Tong University Affiliated Sixth People’s Hospital, Shanghai, China; ^2^Department of Endocrinology and Metabolism, Shanghai 10th People’s Hospital, School of Medicine, Tongji University, Shanghai, China

**Keywords:** thyroid hormone, uric acid production, Period2, nucleotide metabolism, purine metabolism

## Abstract

The relationship between thyroid hormones and serum urate is unclear. Our aim is to analyze the correlation between uric acid and thyroid hormones in gout patients and to explore the effect and mechanism of triiodothyronine on liver uric acid production. Eighty men patients with gout were selected to analyze the correlation between blood urate and thyroid function-related hormone levels. Stepwise multiple linear regression was used to analyze factors affecting blood urate in patients with gout. Levels of urate in serum, liver, and cell culture supernatant were measured after triiodothyronine treatment. Purine levels (adenine, guanine, and hypoxanthine) were also measured. Expression levels of Period2 and nucleotide metabolism enzymes were analyzed after triiodothyronine treatment and Period2-shRNA lentivirus transduction. Chromatin immunoprecipitation was used to analyze the effects of triiodothyronine and thyroid hormone receptor-β on Period2 expression. The results showed that in patients FT3 influenced the serum urate level. Furthermore, urate level increased in mouse liver and cell culture supernatant following treatment with triiodothyronine. Purine levels in mouse liver increased, accompanied by upregulation of enzymes involved in nucleotide metabolism. These phenomena were reversed in Period2 knockout mice. Triiodothyronine promoted the binding of thyroid hormone receptor-β to the Period2 promoter and subsequent transcription of Period2. Triiodothyronine also enhanced nuclear expression of Sirt1, which synergistically enhanced Period2 expression. The study demonstrated that triiodothyronine is independently positively correlated with serum urate and liver uric acid production through Period2, providing novel insights into the purine metabolism underlying hyperuricemia/gout pathophysiology.

## Introduction

Hyperuricemia is considered present when the serum urate level exceeds 420 μmol/L (7 mg/dl) due to a purine metabolism disorder and/or there is reduced urate excretion during intake of a normal purine diet. Urate is the product of purine metabolism and is mainly produced in the liver. Clinical studies have shown that urate is related to many hormones in the body, including luteinizing hormone (LH), follicle-stimulating hormone (FSH), estrogen(E_2_), and thyroid hormone (TH) (Mumford et al., 2013; Mu et al., 2018). Thus far, there have been conflicting reports concerning the relationship between thyroid hormone and serum urate. One study demonstrated that patients with hyperthyroidism exhibited significantly higher levels of urate compared with healthy individuals (Giordano et al., 2001). In addition, a study by Chao et al. (2019) showed that blood urate levels were positively correlated with free T3 (FT3) and free T4 (FT4), instead of thyroid-stimulating hormone, in healthy people. However, other studies reported that thyroid function did not affect serum urate (Ashizawa et al., 2010; Ye et al., 2015). To the best of our knowledge, few studies have focused on the relationship between thyroid hormone and serum urate in patients with hyperuricemia or gout.

Multiple enzymes are involved in the processes of purine and nucleotide metabolism, via which urate is formed, including ribose-phosphate pyrophosphokinase (PRPS), hypoxanthine-guanine phosphoribosyl transferase (HPRT) and xanthine oxidation, adenine phosphoribosyl-transferase (APRT), adenosine deaminase (ADA), and nucleoside phosphorylase (PNP) (Zhang et al., 2017; Feng et al., 2020). In heart and muscle, thyroid hormones are closely related to purine and pyrimidine nucleotide metabolism. In muscle, thyroid hormone can accelerate the purine nucleotide cycle, thereby compensating for the rapid consumption of glycogen and ATP during exercise (Fukui et al., 2001; Doi et al., 2003; Kung, 2007). In the heart, T4 treatment can affect ADA activity, thereby affecting the levels of adenosine and hypoxanthine, which promotes cardiac hypertrophy (Smolenski et al., 1995). In the liver, studies of thyroid hormone have mainly focused on the metabolism of lipids, cholesterol, and glucose (Mullur et al., 2014; Sinha et al., 2018). It is not yet clear whether thyroid hormone affects the production of urate in the liver, or whether thyroid hormone affects the expression of enzymes involving nucleotide metabolism.

Furthermore, the enzymes involved in the metabolism of purine nucleotides to form uric acid have also been reported to be regulated by circadian rhythm gene. Research by Fustin et al. (2012) showed that circadian rhythm gene Bmal1 had a pervasive influence on hepatic nucleotide metabolism and Bmal1^–/–^ mice showed low nucleotide abundance in liver. In addition, Eckel-Mahan et al. (2012) reported that the metabolites, including nucleotide metabolism, is controlled by circadian rhythms gene Clock by mass spectrometry. Period-2 (Per2) is one of the core circadian clock genes and several studies showed that thyroid hormone can regulate Per2 expression in pituitary and gonadal tissue (Amir and Robinson, 2006; De Bargi-Souza et al., 2019). At present, whether thyroid hormone can affect Per2 expression in liver and therefore affect nucleotide metabolism is not yet clear.

Therefore, this study analyzed the relationships of thyroid function-related hormones with serum urate levels in male patients with gout. It also performed a mechanistic analysis of the effects of triiodothyronine (T3) on urate production *in vitro* and *in vivo*.

## Materials and Methods

### Patients and Clinical Data

We performed a case-control study to investigate the relationship between thyroid hormones and serum urate in patients with gout.

Inclusion criteria: (1) all the enrolled 80 male patients were 20 and 50 years old; (2) all the patients were diagnosed with gout based on the 2015 American College of Rheumatology/European League Against Rheumatism diagnostic criteria in the department of endocrinology and metabolism in Sixth People’s Hospital affiliated to Shanghai Jiao Tong University (Shanghai, China) from March 2019 to September 2019. Exclusion criteria: exclude patients with secondary hyperuricemia, thyroid disease, malignant tumors, mental illness, or serious diseases of physiological systems. Informed consent was obtained from all participants.

Clinical parameters including alanine aminotransferase (ALT), aspartate aminotransferase (AST), triglyceride (TG), cholesterol (TC), serum urate, blood urea nitrogen (BUN), fasting blood glucose, glycosylated hemoglobin, thyroid function-related hormones and so on were collected at 8:00 am in the morning. In addition, 24-h urine sample (from 8:00 am-8:00 am) was obtained to detect glomerular filtration rate and uric acid excretion indicators. The study was approved by the Ethics Committee of the Sixth People’s Hospital affiliated to Shanghai Jiao Tong University (Approval No. 2018-102).

### Cell Culture

HepG2 cells and Hep1-6 cells were purchased from the Cell Resource Center (SIBS) of the Shanghai Institute of Biological Sciences. The culture conditions are High Glucose DMEM medium (GIBCO, United States) containing 10% fetal bovine serum, 100 U/mL penicillin and 100 ug/mL streptomycin, in a 37°C, 5% CO_2_ cell condition.

### Treatment and Uric Acid Analysis

The cells were seeded into a 24-well plate at a density of 2 × 10^4^ cells per well. After treatment, collect the cell culture supernatant. The concentration of uric acid was detected by the uric acid detection kit (K608, Biovision, United States) according to the operating instructions. In general, mix 50 μl of cell culture supernatant with reaction mix of the kit, and measure at OD 570 nm for uric acid assay in a microplate reader after 30 min incubation at 37°C. Finally, the total cell protein was used for normalization.

The dose of T3 (T3, Sigma, United States) range from 100 to 1000 nM and was treated for 48 h before uric acid analysis. The dose of EX527 (MCE, China) was 10 μM and was pre-treated for 6 h before T3 was treated. For serum shock, cells were subjected to 50% serum for 2 h and then treated with T3 for 48 h.

### RNA Extraction and Real-Time Quantitative RT-PCR (qPCR) Analysis

Total RNA was isolated using Trizol method (Invitrogen, United States). The obtained RNA (1000 ng) was reverse transcribed into cDNA by HiScript III RT SuperMix for qPCR (Vazyme, China): stage 1: 50°C, 15 min; stage 2: 85°C, 5 s. Use AceQ Universal SYBR qPCR Master Mix (Vazyme, China) to perform qPCR reaction on LightCycler480 (Roche, United States). cDNA from mice livers was diluted 10 times and cDNA from cells was diluted five times. The primer sequences have been listed in Supplementary Tables 1, 2, and *Actb* was chose as the housekeeping genes to normalize the qPCR results.

### Western Blot

RIPA lysis buffer (Beyotime, China) was used to collect protein samples. Nuclear and cytoplasm samples were harvested with NE-PER Nuclear and Cytoplasmic Extraction Reagents (Thermo, United States) according to the manufacturer’s instruction. Then the collected samples (total 30 μg protein) were subjected to sodium dodecyl sulfate polyacrylamide gel electrophoresis (SDS-PAGE) for electrophoretic separation. Then, the samples were transferred to polyvinylidene fluoride (PVDF) membrane (Millipore, United States), following with blocking. Antibodies were Incubated overnight at 4°C: Period2 (ab227727, Abcam, United States, 1:1000), ADA (13328-1-AP, Proteintech, China, 1:1000), ADSL (15264-1-AP, Proteintech, China, 1:1000), APRT (21405-1-AP, Proteintech, China, 1:1000), PRPS1 (15549-1-AP, Proteintech, China, 1:1000), PRPS2 (27024-1-AP, Proteintech, China, 1:1000), PNP (ab244255, Abcam, USA, 1:1000), Actin (#8457, CST, United States, 1:1000), Sirt1 (ab189494, Abcam, United States, 1:1000, RRID:AB_2864311, GAPDH (#5174, CST, United States, 1:1000, RRID:AB_10622025 and Histone 3A (ab1791, Abcam, United States, 1:2000, RRID:AB_302613). HRP-labeled goat anti-mouse antibody and goat anti-rabbit antibody (Beyotime, China, 1:1000) were incubated for 1 h at room temperature. Use protein chemiluminescence HRP substrate kit (Millipore, United States) to detect protein expression.

### Immunohistochemistry

The samples were fixed in 4% paraformaldehyde and then embedded in paraffin. After dewaxing, the section was stained with anti-Period2 antibody (ab227727, Abcam, United States, 1:500), PRPS1 (15549-1-AP, Proteintech, China, 1:500), PRPS2 (27024-1-AP, Proteintech, China, 1:500), ADSL (15264-1-AP, Proteintech, China, 1:250), APRT (ab196558, Abcam, United States, 1:250), ADA (13328-1-AP, Proteintech, China, 1:250), PNP (ab244255, Abcam, United States, 1:250) overnight at 4°C. The next day, horseradish peroxidase-labeled secondary antibody were incubated for 1 h at room temperature. Use an optical microscope (Leica, Germany) to observe the expression of target protein after diaminobenzidine (DAB) was incubated.

For cells, the cells were fixed with 4% paraformaldehyde after 48h of treatment, they were washed with PBS (GIBCO, United States) and permeabilized with 0.2% Triton-X (Sigma, United States) for 15 min at room temperature. The cells were then blocked in 5% BSA (Beyotime, China) for 60 min. Incubate the antibody (Sirt1, ab189494, Abcam, United States, 1:250) overnight at 4°C. The next day, the cells were washed and incubated with the secondary antibody (Beyotime, China, 1:1000) and DAPI (Beyotime, China) according to the protocol, the images were obtained by the fluorescence microscope (Leica, Germany).

### Cell Transfection and Dual Luciferase Reporter Assay

The cells were seeded into a 24-well plate, and transfected with Per2-promoter-PGL3-basic plasmid and Renilla plasmid (Asia, China), siPer2 [GenePharma, China, the sequence of siPer2 (Gery et al., 2007) is: 5′-GTGAACAGCCGCACGGGA-3′] or Per2 overexpression plasmid (GenePharma, China) with green fluorescence protein (GFP) using Lipofectamine 2000 transfection reagent (Invitrogen, United States) according to the instructions. After 48 h, 20 μl (100 μl total) cell lysate was analyzed by the dual luciferase kit was used (Promega, United States) according to the operating instructions and the transfection efficiency was normalized by determining the Renilla luciferase activity. For western blot analysis, protein samples was prepared after 48 h of transfection.

### ChIP Assay

DNA samples were obtained using SimpleChIP^®^ Enzymatic Chromatin IP Kit (9002, CST, United States) according to the instructions, and anti-thyroid hormone receptor beta (ab5622, Abcam, United States, 1:50, RRID:AB_304991) antibody was used for immunoprecipitation. Per2 promoter specific primers: Primer1: forward 5′-AGTCGCCGCTGTCACATAG-3′, reverse 5′-AGGAACCGACGAGGTGAAC-3′; Perimer2: forward 5′-AAGTTGTCCTTGTCTCACC-3′, reverse 5′-AGG AACCGACGAGGTGAAC-3′; Primer3: forward 5′-GGGTG TCCACTCTTTGGT-3′, reverse 5′-CCTGGTCTATGTCTGG GA-3′.

### Animal Experiment

Eight-week-old male C57 mice purchased from Shanghai Experimental Animal Center (Shanghai, China), raised in the room with air conditioning at a temperature of 23–25°C, and a light/dark cycle of 12 h/12 h [light on at 8:00 in the morning (ZT0) and light off at 20:00 in the evening (ZT12)]. The mice can obtain food and water freely and all mice were given oxonic acid (250 mg/Kg body weight/d) by gavage, which can inhibit urate degradation *in vivo*. All experiments were approved by the Animal Experimentation Committee of The Sixth People’s Hospital affiliated to Shanghai Jiao Tong University (Approval No. 2020-0088).

Firstly, select mice (*n* = 8/group) for control group and triiodothyronine group. In triiodothyronine group, triiodothyronine (0.1 μg/g body weight) (Talhada et al., 2019), was intraperitoneally injected every other day at 9:00 am (ZT1). Control group was intraperitoneally injected with PBS. After 2 weeks of treatment, the mice were euthanized for further study.

Secondly, select 60 mice and divide into three groups: control group, triiodothyronine group and triiodothyronine + Per2-shRNA lentivirus vector (LV-shPer2, Asia, China) group. LV-shPer2 was injected through tail vein 7 days before triiodothyronine treatment. Animals (*n* = 5/group) were sacrificed every 6 h (at ZT3, ZT9, ZT15, and ZT21) under a safe red light.

### Liquid Chromatography-UV Analysis

The chromatographic separation was achieved using a U3000-Right system (Thermo, United States) with a UV detector. In general, 300 mg liver sample was triturated in tissue disrupter and was diluted (1:1.25, v/v) with 10% perchloric acid. Then samples were subjected to centrifugation (12000 rpm for 10 min) at room temperature. The supernatant was adjusted to pH = 7.4 and stored at 4°C for analysis. The analysis was carried out using an octadecyl bonded silica column (Agilent Technologies, Germany). During the analysis, the mobile phase was ultrapure water: methanol (55:45, v/v), the flow rate was 1.0 mL/min, and the injection volume was 10 μL. The detection wavelength is 260 nm and the total running time is 25 min.

### Statistical Processing

To ensure repeatability, the experiment was repeated three times. The measurement data was expressed in Mean ± SD. Student’s *t*-test is used to compare the means of two groups, one-way analysis of variance is used for multiple groups. The stepwise logistic regression method was used to analyze the correlation among multiple factors by SPSS 23.0 statistical software. *P* < 0.05 is considered statistically significant.

## Results

### Serum Urate Is Associated With Thyroid Hormone Level in Patients With Gout

**FIGURE 1:**
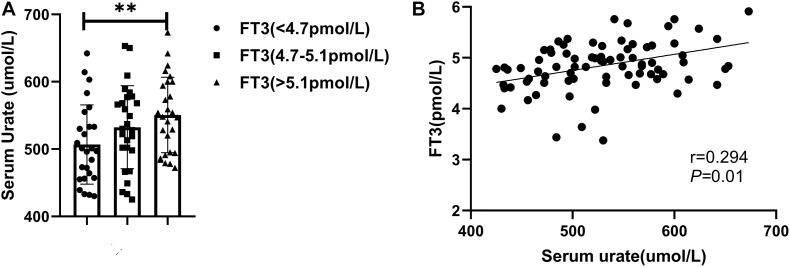
**(A)** Patients were divided into three groups according to FT3 tertiles (^∗∗^*P* < 0.01). **(B)** A positive correlation was observed between FT3 and serum urate level.

To investigate the relationship between serum urate and thyroid function-related hormones, we recruited 80 men patients with gout who had no history of thyroid disease. The mean patient age was 38.0 ± 7.3 years. The mean serum urate level was 530.3 ± 69.4 μmol/L and the mean FT3 level was 4.9 ± 0.5 pmol/L. Other clinical parameters are shown in Supplementary Table 3. The 80 patients were divided into three groups, according to FT3 tertiles. As shown in Figure 1, patients with higher FT3 levels had higher serum urate levels, and FT3 was positively correlated with serum urate (Figure 1 and Supplementary Table 4). In addition, serum urate level was correlated with body mass index (BMI; *r* = 0.292, *P* < 0.01), systolic blood pressure (SBP; *r* = 0.258, *P* < 0.05), alanine aminotransferase (ALT; *r* = 0.264, *P* < 0.05), gamma-glutamyl transpeptidase (rGT; *r* = 0.309, *P* < 0.01), and C-reactive protein (CRP; r = 0.350, *P* < 0.01). It also exhibited negative correlations with the estimated glomerular filtration rate (eGFR; *r* = –0.284, *P* < 0.05) and fractional excretion of uric acid (FEUA; r = –0.240, *P* < 0.05). Stepwise multiple linear regression was performed with serum urate level as the dependent variable. The independent variables were BMI, SBP, ALT, rGT, eGFR, FEUA, CRP, and FT3. The results (Supplementary Table 5) showed that ALT (β = 0.313, *P* < 0.01), eGFR (β = −0.272, *P* < 0.01), FEUA (β = –0.204, *P* < 0.05), and FT3 (β = 0.300, *P* < 0.05) were independently associated with serum urate level.

### Effect of Triiodothyronine on the Production of Urate in Mouse Liver and Cells

**FIGURE 2:**
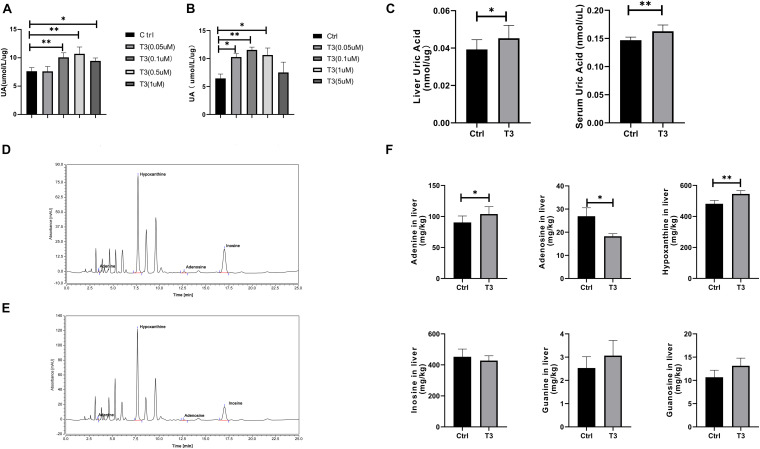
Urate levels in culture supernatant of HepG2 cells **(A)** and Hep 1-6 cells **(B)** at 48 h after treatment. **(C)** Urate levels in mouse liver and serum samples. Liquid chromatography analysis of adenosine, adenine, hypoxanthine, and inosine in the control group **(D)** and T3 treatment group **(E)**. **(F)** Levels of adenosine, adenine, guanosine, guanine, hypoxanthine, and inosine in the livers of control and T3 treatment group mice (^∗^*P* < 0.05; ^∗∗^*P* < 0.01).

The liver is the main source of urate production, so we investigated changes in urate levels in the culture supernatants of T3-treated HepG2 and Hep1-6 cells (Figures 2A,B). The results showed that the urate level in cell culture supernatant increased after T3 treatment. We used a T3 concentration of 0.5 μM for the subsequent cell experiments. Using an *in vivo* experimental protocol, we investigated changes of urate levels in liver and serum samples taken from mice that had received T3 treatment for 2 weeks. Compared with the control group, the experimental group exhibited elevated levels of urate in both liver and serum (Figure 2C).

Urate is the final product of purine catabolism (Tsushima et al., 2013), so we measured the levels of adenosine, adenine, guanosine, guanine, hypoxanthine, and inosine by liquid chromatography-UV. The levels of adenine and hypoxanthine significantly increased, while the levels of adenosine significantly decreased. There were no significant differences in guanine, guanosine, or inosine levels between the two groups (Figures 2D–F).

### Triiodothyronine Regulates the Levels of Enzymes Involved in Nucleotide Metabolism and Period2, Which Affect Urate Production

**FIGURE 3:**
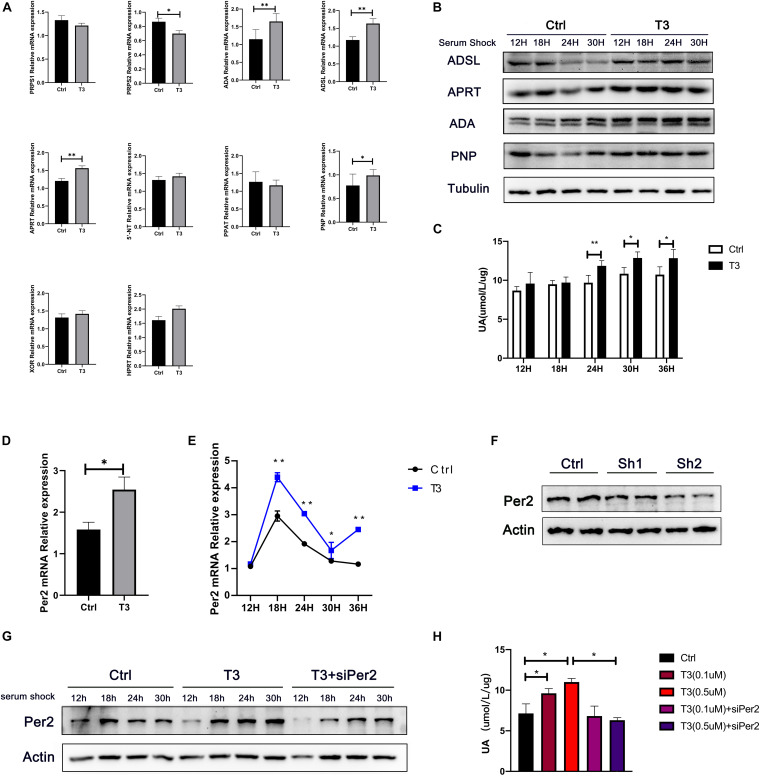
**(A)** mRNA levels of enzymes involved in nucleotide metabolism in mouse liver, Actb was chose as the housekeeping genes to normalize the qPCR results. **(B)** Protein levels of ADSL, APRT, PNP, and ADA in HepG2 cells after serum shock. **(C)** Urate levels in cell culture supernatant at different points after serum shock. **(D)** Per2 mRNA levels in liver after T3 treatment, Actb was chose as the housekeeping genes to normalize the qPCR results. **(E)** Per2 mRNA levels in HepG2 cells at different time points after serum shock, Actb was chose as the housekeeping genes to normalize the qPCR results. **(F)** Protein levels at 48 h after transfection with siPer2. **(G)** Per2 levels in HepG2 cells after serum shock. **(H)** Urate production in HepG2 cells at 48 h after treatment (*P < 0.05; **P < 0.01).

We found that T3 can increase the levels of adenine, hypoxanthine, and urate in mouse liver, suggesting that T3 may affect purine metabolism and urate production. Therefore, we investigated changes in the levels of multiple enzymes involved in purine metabolism in the liver. The results showed that T3 can affect the mRNA and protein levels of many enzymes, including PRPS2, ADA, adenylosuccinate lyase (ADSL), APRT, PNP, and HPRT (Figure 3A and Supplementary Figure 1). PRPS participate in the synthesis of phosphoribosyl pyrophosphate (PRPP). ADA, ADSL, and APRT participate in the purine nucleotide cycle, promoting the formation of AMP, IMP, and inosine. PNP participates in the formation of hypoxanthine. Notably, increases in the hypoxanthine level promote urate formation, catalyzed by xanthine oxidation (XO).

Additionally, we analyzed urate levels in cell supernatants at different time points after T3 treatment, as well as the levels of multiple enzymes involved in nucleotide metabolism (Figures 3B,C). In the T3 treatment group, the levels of ADSL increased at 24 and 30 h after serum shock; APRT increased at 12 and 24 h after serum shock; ADA increased at 12, 18, and 24 h after serum shock; and PNP increased at 18 and 24 h after serum shock. Concurrently, the levels of urate in cell culture supernatant were elevated at 24, 30, and 36 h after serum shock compared with the control group.

T3 has been shown to affect the expression of the rhythm gene Period2 (Per2) in various peripheral tissues, including pituitary, thyroid, heart, and ovary (Preitner et al., 2002; Mannic et al., 2013; Peliciari-Garcia et al., 2016; De Bargi-Souza et al., 2019). We hypothesized that T3-induced cellular urate production might be related to the expression of Per2. Therefore, we analyzed whether T3 could regulate Per2 expression. We found that Per2 mRNA levels were elevated in mouse liver (Figure 3D and Supplementary Figure 2). Furthermore, Per2 mRNA and protein levels were elevated at 18, 24, and 30 h after serum shock in T3-treated cells (Figures 3E,G and Supplementary Figure 3). siPer2 was used to clarify the relationship between Per2 expression and intracellular urate production. The results showed that siPer2 treatment could significantly reduce Per2 levels in cultured cells (Figures 3F,G). Further analysis of urate levels in cell culture supernatant revealed that T3-induced urate production was reduced by siPer2 transfection (Figure 3H).

### Period2 Overexpression Promotes Urate Production in HepG2 Cells

**FIGURE 4:**
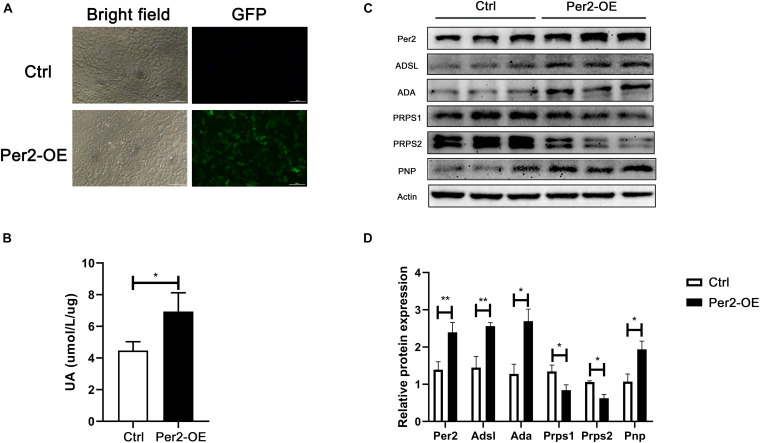
**(A)** Detection of GFP by fluorescence microscopy at 48 h after transfection (Per2-OE, Per2 overexpressing cells). **(B)** Urate production in cell culture supernatant at 72 h after transfection. **(C)** Protein expression after Per2 overexpressing in cells. **(D)** ImageJ (NIH) semiquantitative analysis of the levels of multiple enzymes involved in nucleotide metabolism (*P < 0.05; **P < 0.01).

To investigate whether Per2 can influence nucleotide metabolism enzyme levels and urate production, we transfected a Per2 overexpression plasmid into HepG2 cells (Figure 4A). After 48 h, urate production and Per2 levels were measured. The results showed that urate levels increased in cell culture supernatant after induction of Per2 overexpression. In addition, the levels of ADSL, ADA, and PNP increased, while the levels of PRPS1 and PRPS2 decreased (Figures 4B–D).

### Triiodothyronine Regulates the Expression of Purine Metabolism Enzymes and Urate Production Through Period2

**FIGURE 5:**
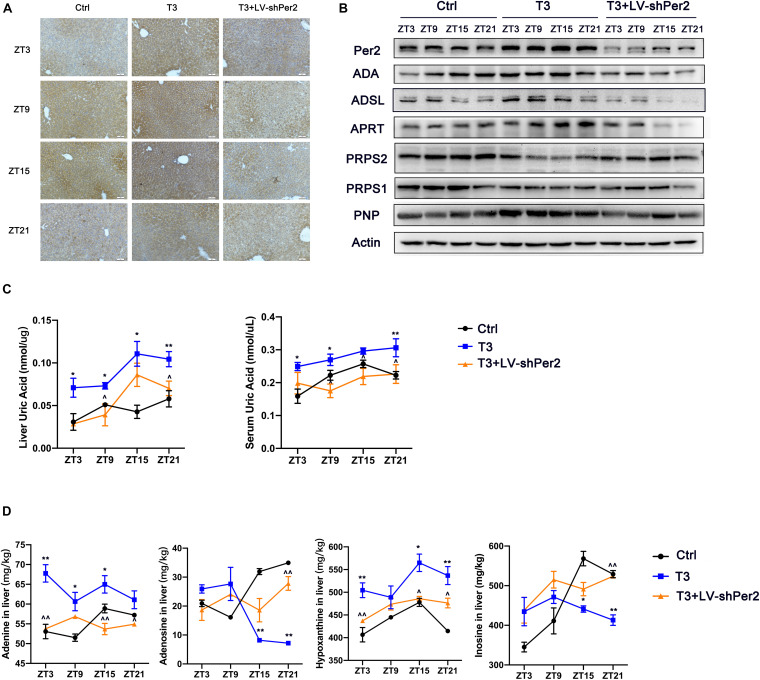
**(A)** Immunohistochemical images of Per2 in mouse liver (200×). **(B)** Expression of nucleotide metabolism enzymes in mouse liver. **(C)** Urate levels in mouse liver and serum samples. **(D)** Levels of adenosine, adenine, hypoxanthine, and inosine in mouse liver (*P < 0.05 and **P < 0.01, T3 group compared with control group; ^*P* < 0.05 and ^^*P* < 0.01, T3 group compared with T3 + LV-shPer2 group).

To verify the effects of T3-induced Per2 expression on nucleotide metabolism enzymes and urate production *in vivo*, we knocked out Per2 expression in mouse liver by lentivirus injection through the tail vein of each mouse. As shown in Figure 5A, western blotting and immunohistochemistry revealed that Per2 levels were significantly reduced in mouse liver after lentivirus injection. The levels of ADSL, ADA, APRT, PRPS1, PRPS2, and PNP were also substantially modified (Figure 5B and Supplementary Figure 4). Compared with the T3 treatment group, liver and serum urate levels were reduced in the T3 + LV-shPer2 group (Figure 5C) at different points. The levels of adenosine, adenine, hypoxanthine, and inosine were also altered in liver tissue (Figure 5D). Finally, the levels of adenine and hypoxanthine were significantly reduced after Per2 had been knocked out, suggesting that Per2 inhibition can reduce T3-induced urate production in the liver.

### TR Binds to the Period2 Promoter to Regulate Its Expression

**FIGURE 6:**
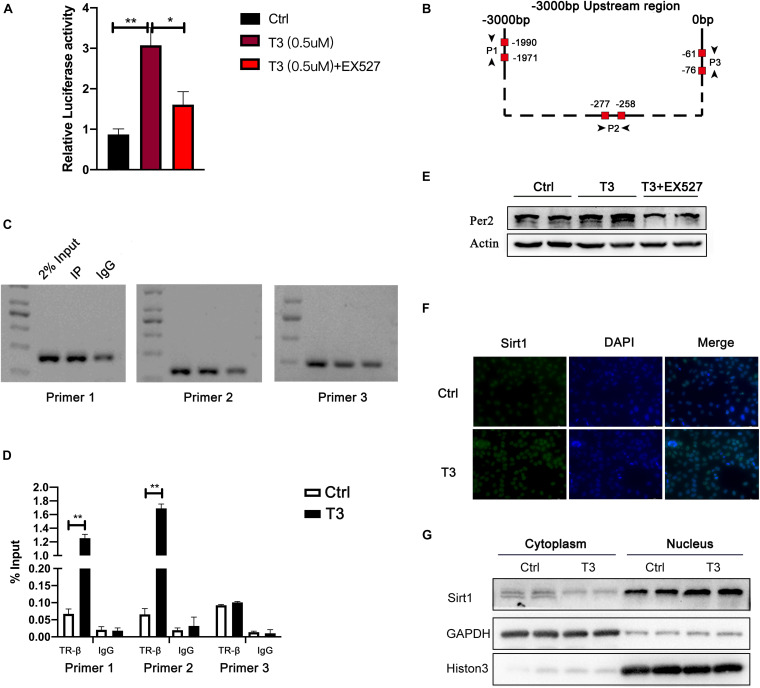
**(A)** Luciferase reporter activity at 48 h after treatment. **(B)** Per2 gene upstream region and TR-β binding site analysis. **(C)** For the predicted binding site, samples of input, TR-β IP, and IgG IP were analyzed by PCR. **(D)** ChIP-qPCR results (**P < 0.01). **(E)** Effect of EX527 on T3-induced Per2 expression. **(F)** Sirt1 immunofluorescence analysis. **(G)** Nuclear levels of Sirt1 in HepG2 cells treated with T3 for 48 h.

T3 can activate thyroid hormone receptor (TR). TR then acts as a transcription factor to promote the transcription of target genes. The most widely expressed TR is TR-β (Sinha et al., 2018). Therefore, we investigated whether T3 directly regulates Per2 expression by promoting the binding of TR-β to the Per2 promoter. First, we conducted luciferase reporter activity analysis and observed the effect of T3 treatment on fluorescence values. The results are shown in Figure 6A. Compared with the control group, T3 treatment led to greater Per2-LUC reporter activity. We then investigated whether TR-β could bind to the Per2 promoter region. We predicted two binding sites (Primer 1 and Primer 2) within 3 kb before the annotated transcription start site of the Per2 gene through hTFtarget and JASPAR website (Figure 6B). We then used an anti-TR-β antibody for ChIP analysis. The results are shown in Figures 6C,D. We were able to specifically amplify DNA fragments from the TR-β samples, but not the IgG samples. Also, there was no binding found on the irrelevant region of Per2 promoters (Primer 3). The qPCR results also showed that T3-activated TR-β can bind to the Per2 promoter.

In addition, several studies have shown that Sirt1 can enhance the expression of a variety of T3-target genes, including peroxisome proliferator-activated receptor coactivator α (PGC-1α), carnitine palmitoyl transferase (CPT1), pyruvate dehydrogenase kinase 4 (PDK4), and phosphoenolpyruvate carboxy kinase (PEPCK) (Suh et al., 2013; Thakran et al., 2013). Here, we hypothesized that Sirt1 might be involved in T3-induced expression of Per2. The results showed that EX527 was able to reduce the fluorescence value of T3-induced luciferase activity and the level of Per2 protein expression in cells (Figures 6A,E). Although the total expression of Sirt1 did not change after T3 treatment, Sirt1 expression increased in the nucleus (Figures 6F,G and Supplementary Figure 5).

## Discussion

Our study investigated the relationship between thyroid hormone levels and liver urate production, and explored the mechanism underlying this relationship. In the cross-sectional analysis, we found that FT3 was positively correlated with serum urate level, suggesting that FT3 influences the production of serum urate. In the animal and cell experiments, we found that the levels of urate in mouse liver and serum were elevated after T3 treatment, accompanied by elevated levels of substances such as adenine and hypoxanthine in the liver. In addition, T3 can affect the levels of multiple enzymes that participate in nucleotide metabolism, thereby promoting the production of urate. Finally, T3 induced changes in the levels of enzymes and purines, partly through TR-β mediated changes in Per2 expression.

Several clinical studies have focused on the relationship between thyroid hormone and blood urate (Raber et al., 1999; Saini et al., 2012; Minami et al., 2015; Ye et al., 2015). Some studies have shown that the serum urate level is positively associated with the thyroid hormone level (Minami et al., 2015; Ye et al., 2015). However, other studies demonstrated no association, or a negative association, between these parameters (Raber et al., 1999; Saini et al., 2012). Our study showed that higher urate levels were associated with higher FT3 levels in patients with gout, and that T3 independently influenced blood urate levels. Furthermore, Sato et al. (1995) showed that in patients with hyperthyroidism, both the blood urate level and renal urate excretion were enhanced. In addition, studies in healthy people have shown that FT3 is directly related to blood urate, suggesting that T3 can promote urate production. The level of blood urate in the body is affected by both production and excretion processes. Our study showed no significant differences in urate excretion among patients grouped according to FT3 tertiles (Supplementary Table 6). Notably, urate levels increased as FT3 levels increased, implying that T3 influences urate production.

The effect of thyroid hormone on nucleotide/purine metabolism has primarily been studied in muscles. During exercise, ATP is consumed rapidly; T3 promotes the acceleration of purine catabolism, which leads to rapid conversion of AMP into IMP and maintenance of energy balance (Fukui et al., 2001). In addition, in the mitochondria of skeletal and cardiac muscles, T3 promotes the expression of adenine nucleotide translocase and production of ATP (Grant, 2007). Among the enzymes involved in nucleotide metabolism, ADA, ADSL, APRT, and PNP participate in the formation of AMP, IMP, inosine, and hypoxanthine. PRPS1 and PRPS2 participates the production of PRPP. Reduced expression of PRPS may induced hypoxanthine accumulation in the purine salvage pathways. Excess hypoxanthine and IMP in the liver can be converted into urate through a process catalyzed by XO (Camici et al., 2018). Zhu et al. (2017) showed that extracts from green tea and black tea were able to inhibit ADA activity, thereby reducing serum urate levels. Furthermore, PNP catalyzes the conversion of inosine into hypoxanthine. PNP inhibitors have been shown to reduce the levels of urate and hypoxanthine in the culture medium of skin fibroblasts from patients with Lesch-Nyhan disease. This finding implies that changes in the activity or expression of enzymes involving nucleotide metabolism can affect urate production in the liver (Jacomelli et al., 2019). Our study found that T3 can promote ADA and PNP expression in liver/hepatocytes, which may contribute to the enhanced production of urate in liver tissue and hepatocytes. ADSL and APRT catalyze the production of AMP. Increased levels of these enzymes may further promote the conversion of AMP into IMP and hypoxanthine.

The relationship between T3 and Per2 has been reported in many tissues. And our results concerning the effect of T3 on up-regulation of Per2 levels in liver and cells were consistent with the findings of prior studies. However, the mechanism of Per2 on uric acid or nucleotide metabolism is not yet clear and needs further research. Biochemical modulation mechanisms of Per2 on metabolism can be summarized into two parts: Per2 not only affects the transcription and expression of target genes by repressing Bmal1-Clock complex, but can extend its regulatory capacity by interact with transcription factors such as cell nuclear receptors (Schmutz et al., 2010). On the one hand, it was reported that, in Bmal1^–/–^ or Clock^–/–^ mice, urate level at CT4 and CT16 or xanthine level elevated in liver (Eckel-Mahan et al., 2012; Fustin et al., 2012). On the other hand, Per2 can bind to hepatocyte nuclear factor (HNF-4α or HNF-1α) or hypoxia-inducible factor 1α (HIF-1α) in liver and heart to influence their transcription activity. The mutation HNF-4α or HNF-1α gene was reported as the cause of MODY, which has complications of hyperuricemia or gout (Schmutz et al., 2010; Firdous et al., 2018). As for HIF-1α, it was reported that Per2 can promote the recruitment of HIF-1α to promoter regions of its downstream genes and HIF-1α participate the nucleotide metabolism through up-regulation expression or activity of enzymes including HPRT, AMP deaminase (AMPD) and XO in heart and inflammatory reactions (Nicholas et al., 2011; Wu et al., 2015). In our study, Per2 knockout was able to reverse changes in the expression of nucleotide-metabolizing enzymes. It also reversed T3-induced increases in the levels of hypoxanthine and urate. Moreover, the levels of PRPS2, ADSL, ADA, and PNP were elevated in Per2-overexpressing cells, similar to the effect of T3. Taken together, these findings indicate that T3 can affect liver nucleotide metabolism and urate production by regulating Per2 expression.

In the classical pathway, thyroid hormone binds to TR, which then serves as a transcription factor recognizing thyroid hormone response elements (TREs) in the promoter regions of target genes (Sinha et al., 2018; Bianco et al., 2019). Ligand-dependent receptor recruitment and chromatin remodeling of thyroid hormone plays important role in T3 induced transcriptional activation and there are more than twice as many ligand-facilitated TR binding sites were found after T3 treatment (Grøntved et al., 2015). T3 can upregulate Per2 levels in various tissues, but it has been unclear whether T3 regulates Per2 levels through the T3/TR pathway. Other than BMAL1-CLOCK complex, it was reported that many regulatory elements exist in the Per2 promoter region, including steroid hormone response elements, cAMP response elements, and D-box elements, implying that its expression may be affected by various hormones (Nakao et al., 2007; Chu et al., 2012). Here, ChIP analysis of hepatocytes showed that TR-β was able to bind to the Per2 promoter region, indicating that T3 regulates Per2 levels through the T3/TR pathway.

Finally, Sirt1 is known to regulate Per2 expression. Asher et al. (2008) showed that Sirt1 binds to the CLOCK-BMAL1 complex in a circadian manner, during which it promotes the deacetylation and degradation of Per2. Chang and Guarente (2013) found that overexpression of Sirt1 promoted the production of Bmal1, Per2, Cry1, and RORα by activation of PGC-1α. The role of Sirt1 may different among tissue and cell types. Sirt1 and resveratrol have both been shown to cooperate with T3, thereby enhancing the transcription-promoting effect of T3 on target genes (Singh et al., 2013; Suh et al., 2013; Thakran et al., 2013). Our analysis showed that T3 was able to directly regulate Per2 transcriptional levels through TR-β binding to the Per2 promoter. In addition, a Sirt1 inhibitor, EX527, was able to inhibit the T3-mediated induction of Per2.

## Conclusion

In conclusion, T3 was able to influence the levels of enzymes involved in nucleotide metabolism in the liver, and promoted urate production by inducing Per2 expression, which providing novel insights into the relationship between thyroid hormone and serum urate and the underlying mechanism in hyperuricemia/gout pathophysiology.

## Data Availability Statement

The original contributions presented in the study are included in the article/Supplementary Material, further inquiries can be directed to the corresponding author/s.

## Ethics Statement

The studies involving human participants were reviewed and approved by the Ethics Committee of the Sixth People’s Hospital affiliated to Shanghai Jiao Tong University (Approval No. 2018-102). The patients/participants provided their written informed consent to participate in this study. The animal study was reviewed and approved by the Animal Experimentation Committee of The Sixth People’s Hospital affiliated to Shanghai Jiao Tong University (Approval No. 2020-0088).

## Author Contributions

HC and SQ designed the research. XC and MW performed the research. NL and JL collected and analyzed the data. XC wrote the manuscript. HC reviewed and edited the manuscript. All the authors contributed to the manuscript and gave their final approval of the version to be published and then wrote and edited the manuscript.

## Conflict of Interest

The authors declare that the research was conducted in the absence of any commercial or financial relationships that could be construed as a potential conflict of interest.
