# Supplementary material for: Thyroid Hormone-Regulated Expression of Period2 Promotes Liver Urate Production
Source: Front Cell Dev Biol. 2021 Apr 1;9:636802. doi: 10.3389/fcell.2021.636802 (PMC8047155; doi:10.3389/fcell.2021.636802)
Supplement: Supplementary file 1 [file Data_Sheet_1.docx]

**Supplementary information:**

**Figures:**


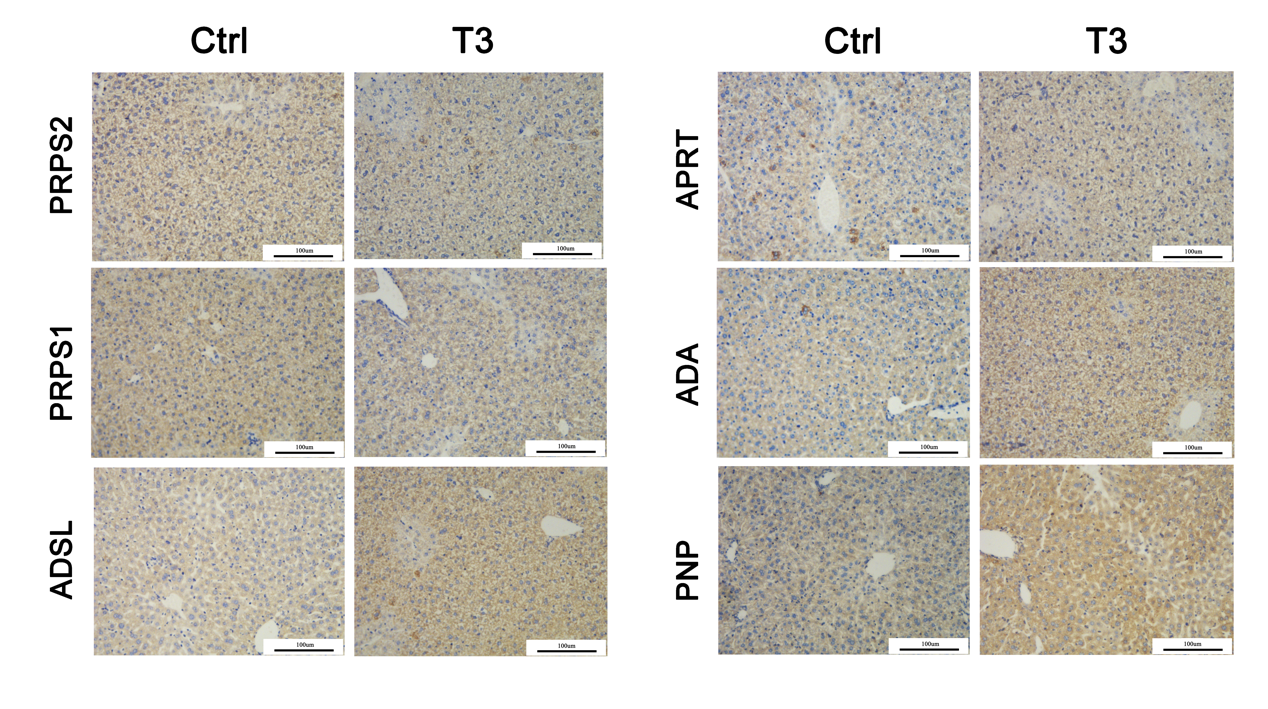


Figure S1. Histochemical staining of enzymes involving nucleotide metabolizing in mice liver.


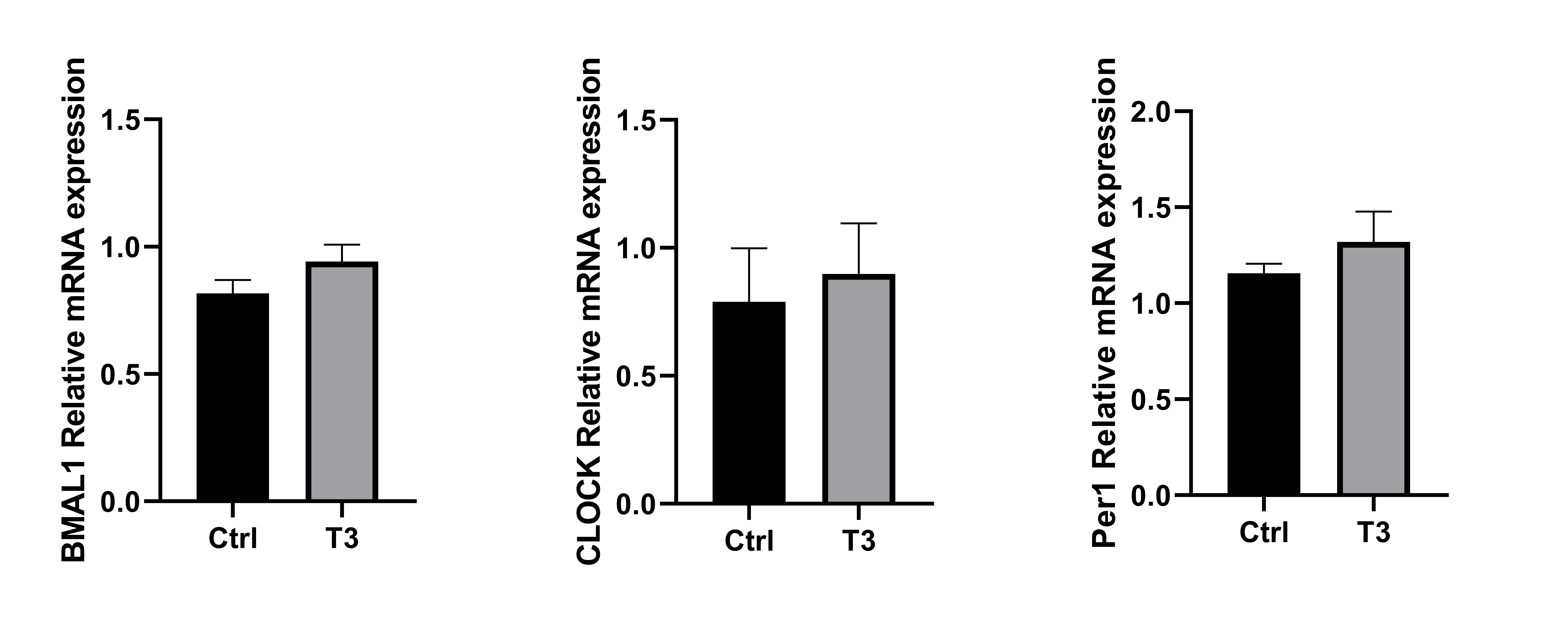


Figure S2. mRNA expression of BMAL1, CLOCK and Per1 in mouse liver.


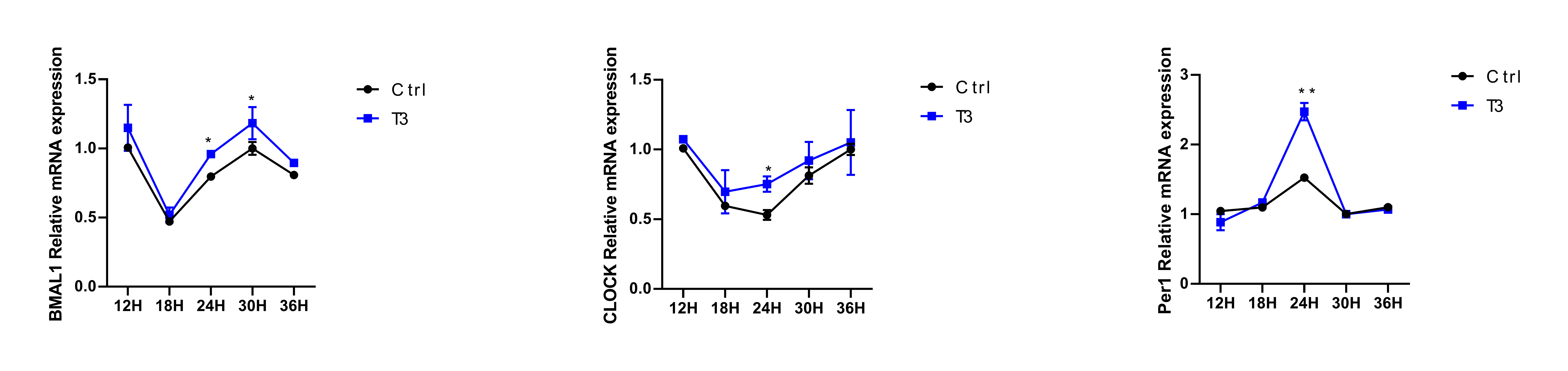


Figure S3. mRNA expression of BMAL1, CLOCK and Per1 in HepG2 cells at different times after serum shock.


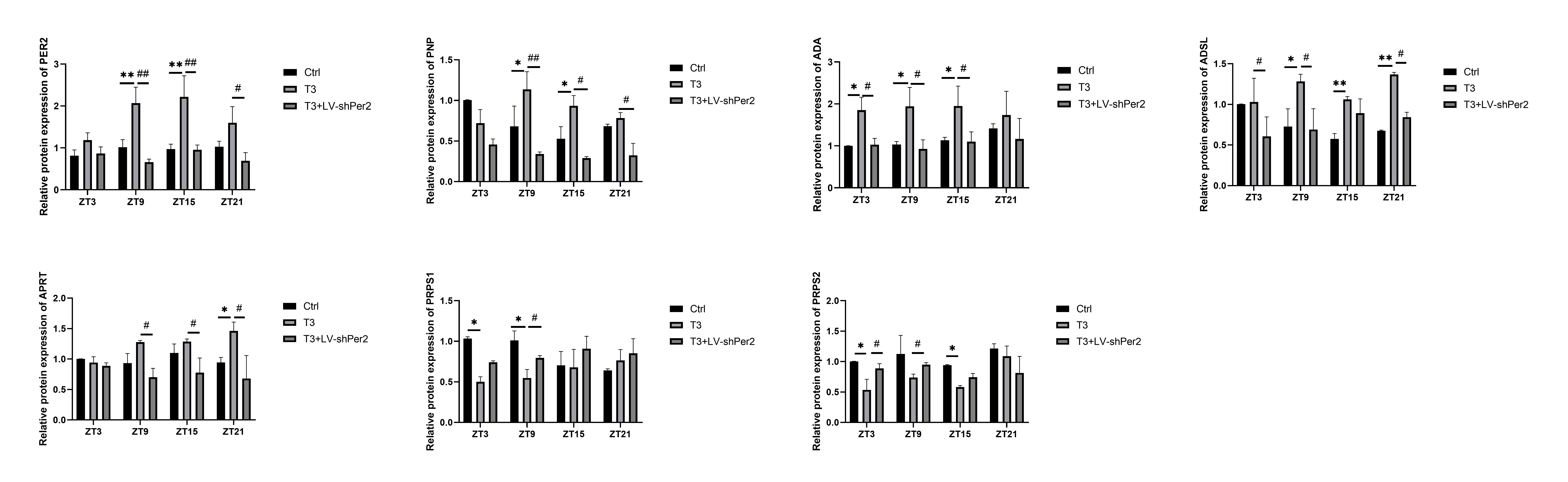


Figure S4. ImageJ (NIH) semiquantitative analysis of the levels of multiple enzymes involved in nucleotide metabolism (*/^#^,P<0.05; **/^##^,P<0.01)


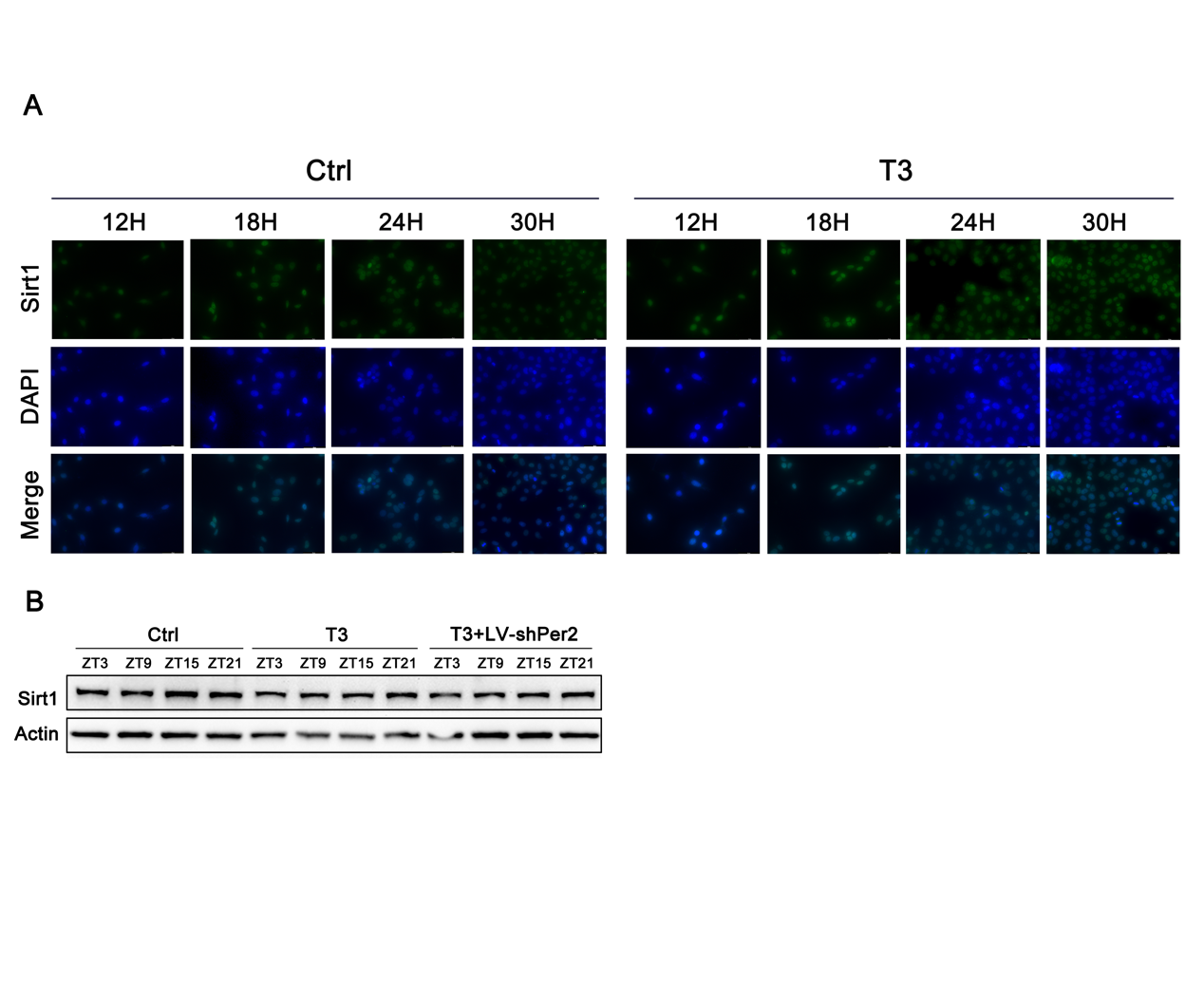


Figure S5. (A) Immunofluorescence staining of sirt1 in HepG2 cells after serum shock. (B) Protein expression of Sirt1 in mice liver.

**Tables:**

Table S1. Full names and abbreviations of enzymes

| Enzymes | Abbreviations |
| --- | --- |
| Xanthine oxidation | *XOR* |
| Ribose-phosphate pyrophosphokinase | *PRPS* |
| Hypoxanthine-guanine phosphoribosyl transferase | *HPRT* |
| Adenine phosphoribosyl-transferase | *APRT* |
| Adenosine deaminase | *ADA* |
| Nucleoside phosphorylase | *PNP* |
| Phosphoribosyl pyrophosphate amidotransferase | *PPAT* |
| Adenylosuccinate lyase | *ADSL* |
| 5'-nucleotidase | *5’-NT* |

Table S2. Primers used were for real-time PCR

| Gene | Accession | Forward | Reverse |
| --- | --- | --- | --- |
| **Mouse** |  |  |  |
| PRPS1 | XM_021187567 | ACTTATCCCAGAAAATCGCTGAC | CCACACCCACTTTGAACAATGTA |
| PRPS2 | XM_030572906 | ATGCCTAACATCGTGCTCTTC | GATCTCGACACTGGTCTCCTG |
| ADA | XM_003825933 | CCCTCCCGGCAGATACAGT | AGGCATGTAGTAGTCAAACTTGG |
| ADSL | XM_034517282 | TCTGCCCACGTTAGGTTTCAC | CGCTTCAAGTTCTGGAGATCC |
| APRT | XM_021170527 | CCCTCTTGAAAGACCCGGAC | TCCAGAGAATAGGAGGCTGAC |
| 5’-NT | NM_011851 | CCTGCACACAAACGACGTG | CTGGTCTCCGGCATCCAAAA |
| PPAT | XM_021161829 | TTCAGGGTGCATAAGGGAATGG | GCGTACCTCGTATGTCCGA |
| PNP | XM_032917866 | ATCTGTGGTTCCGGCTTAGGA | TGGGGAAAGTTGGGTATCTCAT |
| XOR | XM_021651204 | ATGACGAGGACAACGGTAGAT | TCATACTTGGAGATCATCACGGT |
| HPRT | NM_013556 | TCAGTCAACGGGGGACATAAA | GGGGCTGTACTGCTTAACCAG |
| Per2 | XM_029476476 | AAAGCTGACGCACACAAAGAA | ACTCCTCATTAGCCTTCACCT |
| Per1 | XM_006532480 | GAATTGGAGCATATCACATCCGA | CCCGAAACACATCCCGTTTG |
| BMAL1 | XM_030242020 | TGACCCTCATGGAAGGTTAGAA | GGACATTGCATTGCATGTTGG |
| CLOCK | XM_017320649 | AGAACTTGGCATTGAAGAGTCTC | GTCAGACCCAGAATCTTGGCT |
| Actin | NM_007393 | GGCTGTATTCCCCTCCATCG | CCAGTTGGTAACAATGCCATGT |
| **Human** |  |  |  |
| PRPS1 | NM_001204402 | ATCTTCTCCGGTCCTGCTATT | TGGTGACTACTACTGCCTCAAA |
| PRPS2 | NM_001039091 | AGCTCGCATCAGGACCTGT | ACGCTTTCACCAATCTCCACG |
| ADA | XM_035298685 | GCCTTCGACAAGCCCAAAGTA | CTCTGCTGTGTTAGCTGGGAG |
| ADSL | NM_001363840 | ACATTGGGTTTGCCTATCACAG | GCCATCACATCATGTCGTAAACG |
| APRT | XM_008974685 | GGCCGCATCGACTACATCG | CTCAGCCTTCCCGTACTCC |
| 5’-NT | NM_001351179 | CTCCTGGAGTGATCGGTTACA | AGGCTTCTCGACGATACTTTTTC |
| PPAT | NM_002703 | GATGGGAGTTCGGTGCCAA | CAACGAAGGGCTGACAATTTTC |
| PNP | NM_000270 | ATGGAGAACGGATACACCTATGA | GAGGTCGGTGCTTAGTGTGAG |
| XOR | XM_033176128 | CTGCGATTTGAAGGGGAGC | TCAATGCCAATCTCCGTGTTC |
| HPRT | XM_035288166 | CCTGGCGTCGTGATTAGTGAT | AGACGTTCAGTCCTGTCCATAA |
| Per2 | NM_022817 | GACATGAGACCAACGAAAACTGC | AGGCTAAAGGTATCTGGACTCTG |
| Per1 | XM_024926042 | ACGGGCCGAATCGTCTACA | TGGAACCATAGAAGACTCCCAC |
| BMAL1 | NM_001351824 | AAGGGAAGCTCACAGTCAGAT | GGACATTGCGTTGCATGTTGG |
| CLOCK | NM_001267843 | CAACACCAACCAAGATCCCGAC | AATGATGACCTTCTTTGCACCA |
| Actin | NM_001101 | CATGTACGTTGCTATCCAGGC | CTCCTTAATGTCACGCACGAT |

Table S3. Clinical data of patients (Mean±SD)

| Clinical characteristics |  |
| --- | --- |
| N | 80 |
| Age, Year | 38.0±7.3 |
| BMI, Kg/m^2^ | 26.4±5.0 |
| WHR | 0.94±0.05 |
| SBP, mmHg | 130.0±16.2 |
| DBP, mmHg | 86.1±12.2 |
| Duration of gout, year | 5.3±6.4 |
| ALT, U/L | 38.3±22.2 |
| AST, U/L | 23.3±7.4 |
| rGT, U/L | 54.3±45.9 |
| TC, mmol/L | 5.1±0.8 |
| TG, mmol/L | 2.7±2.1 |
| HDL, mmol/L | 1.1±0.2 |
| LDL, mmol/L | 3.1±0.8 |
| Serum urate, umol/L | 530.3±69.4 |
| FEUA, % | 9.8±7.2 |
| eGFR, ml/min/1.73m^2^ | 93.6±17.9 |
| Fasting blood glucose, mmol/L | 5.6±0.8 |
| Glycosylated hemoglobin, % | 5.5±0.4 |
| C reactive protein, mg/L | 4.7±9.5 |
| FT3, pmol/L | 4.9±0.5 |
| FT4, pmol/L | 17.0±2.5 |
| TSH, mIU/L | 2.4±1.3 |

Table S4. Pearson correlation analysis of serum uric acid level with other clinical parameters

|  | | **SUA** | |
| --- | --- | --- | --- |
|  | *r* | | *P* |
| BMI, Kg/m^2^ | 0.292 | | 0.009 |
| SBP, mmHg | 0.258 | | 0.022 |
| ALT, U/L | 0.264 | | 0.018 |
| rGT, U/L | 0.309 | | 0.005 |
| eGFR, ml/min/1.73m^2^ | -0.284 | | 0.011 |
| FEUA, % | -0.240 | | 0.036 |
| C reactive protein, mg/L | 0.350 | | 0.002 |
| FT3, pmol/L | 0.294 | | 0.010 |

Table S5. Results of stepwise multiple linear regression analysis for variables associated with serum uric acid

|  | β | std | Standarized Coefficients Beta | *P* |
| --- | --- | --- | --- | --- |
| Constant | 525.950 | 54.393 |  | <0.01 |
| ALT | 0.815 | 0.261 | 0.313 | <0.01 |
| eGFR | -0.938 | 0.345 | -0.272 | <0.01 |
| FT3 | 35.017 | 11.817 | 0.300 | <0.01 |
| FEUA | -2.791 | 1.381 | -0.204 | <0.05 |

Table S6. Serum urate and uric acid excretion in each group

|  | FT3  (<4.7pmol/L) | FT3  (4.7-5.1pmol/L) | FT3  (>5.1pmol/L) |
| --- | --- | --- | --- |
| N | 26 | 27 | 26 |
| CUA, ml/min | 12.3±10.5 | 11.6±9.6 | 11.1±5.8 |
| EUA, mg/24h/kg | 0.14±0.14 | 0.30±0.12 | 0.32±0.10 |
| FEUA, % | 10.8±9.4 | 9.0±6.8 | 8.9±4.0 |
| eGFR, ml/min/1.73m^2^ | 94.9±15.4 | 91.3±18.4 | 94.6±19.8 |
| Serum urate, umol/L | 506.7±69.3 | 533.6±64.2 | 549.4±57.7^a^ |

a, <0.05, compared with FT3 (<4.7pmol/L) group.
